# Serum concentrations of HGF and IL-8 in patients with active Graves’ orbitopathy before and after methylprednisolone therapy

**DOI:** 10.1007/s40618-015-0322-7

**Published:** 2015-06-11

**Authors:** M. Nowak, L. Siemińska, J. Karpe, B. Marek, B. Kos-Kudła, D. Kajdaniuk

**Affiliations:** Pathophysiology Division, Department of Pathophysiology and Endocrinology, School of Medicine with the Division of Dentistry, Medical University of Silesia, pl. Traugutta 2, 41-800 Zabrze, Poland; Endocrinology Division, Department of Pathophysiology and Endocrinology, School of Medicine with the Division of Dentistry, Medical University of Silesia, Zabrze, Poland; Department of Anaesthesiology and Intensive Therapy, School of Medicine with the Division of Dentistry, Medical University of Silesia, Zabrze, Poland

**Keywords:** Graves’ orbitopathy, Hepatocyte growth factor, Interleukin-8, Methylprednisolone treatment

## Abstract

**Introduction:**

Graves’ disease is the most common cause of hyperthyroidism, and orbitopathy is the most frequent extrathyroidal manifestation of Graves’ disease. The aims of this study were as follows: (1) to evaluate the serum concentration of HGF and IL-8 in the blood of newly diagnosed Graves’ disease patients with the first episode of active GO and healthy controls; (2) to estimate the influence of the thyroid function (euthyreosis vs. hyperthyreosis) on HGF and IL-8 blood levels in patients with active GO; (3) to evaluate the influence of intravenous (i.v.) methylprednisolone (MP) pulse therapy and additional oral MP treatment on HGF and IL-8 blood levels in patients with active GO.

**Patients and methods:**

Thirty-nine Graves’ disease patients with the first episode of clinically active GO (Group A) were enrolled in the study. To estimate the influence of the thyroid function on serum concentrations of the studied proangiogenic factors, Group A was divided into Group A I (*n* = 18) in euthyroid and Group A II (*n* = 21) in hyperthyroid stage of Graves’ disease in moderate-to-severe stage of GO. The control group consisted of 20 healthy volunteers age- and sex-matched to the GO group. Concentrations of the studied proangiogenic factors in serum samples were measured by an enzyme-linked immunosorbent assay before (Group A) and after (Group A1) intensive pulse i.v.MP treatment and 1 month after the end of additional oral MP treatment (Group A2).

**Results:**

We found a significant increase in serum concentrations of studied factors in the GO group before immunosuppressive therapy when compared with the control group and decrease after i.v.MP treatment. One month after the end of additional oral MP treatment (Group A2), serum concentrations of HGF and IL-8 still decreased and no significant difference was observed in HGF and IL-8 concentrations when compared with the control group. We did not find the difference in serum concentration of the studied proangiogenic factors between patients in euthyroid and hyperthyroid stage of Graves’ disease before MP therapy.

**Conclusions:**

Serum HGF and IL-8 concentrations are elevated in Graves’ disease patients with active Graves’ orbitopathy as compared to the healthy control group. Successful management of active Graves’ orbitopathy with glucocorticoids is associated with a decrease in HGF and IL-8 serum concentrations.

## Introduction

Graves’ disease is the most common cause of hyperthyroidism, and orbitopathy is the most frequent extrathyroidal manifestation of Graves’ disease [1]. Graves’ orbitopathy (GO) is the complex of periocular and/or ocular manifestations that are often found in patients with Graves’ disease but also, less frequently, in patients with Hashimoto thyroiditis or patients without thyroid abnormalities [2]. Graves’ orbitopathy occurs before, during, or after the onset of hyperthyroidism and, less frequently, in euthyroid or hypothyroid patients. The course of GO can be divided into active (infiltrative) and inactive (fibrotic) phases.

The estimated incidence of GO in the general population is 16 females and 3 males per 100,000 persons annually, with severe forms accounting for no more than 3–5 % of the cases [3]. The female-to-male ratio is 9.3:1 in patients with mild GO, 3.2:1 in patients with moderate, and 1.4:1 with severe GO [4].

Graves’ orbitopathy is an autoimmune disease in which CD4+ and CD8+ T cells, B cells, plasma cells, and macrophages infiltrate the orbital and periorbital soft tissues, which results in tissue remodelling and oedema. Enlargement of the orbital tissue volume occurs through the accumulation of the extracellular matrix, scar-forming myofibroblasts, and/or fat proliferation [5, 6]. Orbital T cells, fibroblasts, adipocytes and perhaps other residential cells may release numerous cytokines, growth factors, and inflammatory mediators, many of which act as potent stimulators of glycosaminoglycan accumulation and oedema formation. Numerous cytokines, such as IL-1α, IL-4, IL-6, IL-8, IL-10, IL-16, TNF-α, CCL2, and CXCL10 were identified in the orbital tissue and serum from patients with GO and are, therefore, proposed to play a significant role in GO pathogenesis [7–10].

The thyroid-stimulating hormone (TSH) receptor (TSHR) is the autoantigen responsible for the hyperthyroidism in Graves’ disease and is considered an important contributor to GO. The major autoantigens in Hashimoto’s disease are thyroid peroxidase (TPO) and thyroglobulin (Tg). However, these antibodies (anti-TPO and anti-Tg) also occur in about 70 % of Graves’ disease patients [11]. Thyroid-stimulating hormone receptor antibodies (TRAb) are present in all patients with Graves’ disease, and the severity and the activity of GO directly correlate positively with the blood TRAb levels [12–14].

It is suggested that insulin-like growth factor-1 receptor (IGF-1R) stimulatory autoantibodies enhance the production of hyaluronan and T cell chemoattractants (IL-16 and CCL5) by orbital fibroblasts and/or recruited fibrocytes [15]. Other autoantigens were also proposed as putative shared antigens, including several eye muscle antigens or acetylcholine receptor [16]. It remains to be established whether autoimmunity to TSHR is the primary event. However, the role of other autoantigens in the development of GO has not been fully understood as yet.

Angiogenesis, formation of new vascular structures from preexisting vessels, occurs in several organs during multiple pathophysiological conditions. Although it is an integral part of tumour progression, angiogenesis is also observed in different inflammatory, fibrotic, and ischaemic diseases [17–19].

A limitation in oxygen supply is an important stimulus for proangiogenic factors expression. Hypoxia promotes the expansion of monoclonal cells, which have lost their ability to induce apoptosis in response to hypoxia [20]. Lower cellular pH and a higher concentration of lactate could induce the release of proangiogenic factors from macrophages and tumour cells [21]. In GO, the restricted orbital compartment is associated with compression of orbital tissue and slow venous return [22]. This may result in a limitation in the oxygen supply, lower pH, and a higher concentration of lactate which could stimulate the proangiogenic factors expression, independently of other mechanisms such as inflammation. Among the mechanical factors that trigger angiogenesis, an important role is played by increased blood flow in the vascular capillary (e.g. hyperthyroidism) [23].

Activators of angiogenesis include vascular endothelial growth factor (VEGF), hepatocyte growth factor (HGF), transforming growth factor beta 1 (TGFβ1), and its receptors, acidic and basic fibroblast growth factor (aFGF, bFGF), insulin-like growth factor I (IGF-I), platelet-derived growth factor (PDGF), nitric oxide (NO), plasminogen activators (uPA—urokinase plasminogen activator), matrix-metalloproteinases (MMPs), heparinases, chymases, tryptase, cathepsin, angiopoietin-1 (Ang-1) and angiopoietin-2 (Ang-2), plasminogen activator inhibitor 1 (PAI-1), angiotensin II, monocyte chemotactic protein 1 (MCP-1), hypoxia-inducible factor 1 alpha (HIF-1 α), IL-8 and IL-1, epidermal growth factor (EGF), prostaglandin (PGE 1, PGE 2, PGF), erythropoietin, histamine, bradykinin, fibrin, heparin, and tumour necrosis factor alpha (TNF α) [23, 24].

According to the current guidelines of the European Group on Graves’ Orbitopathy, euthyroidism should be restored and maintained stably in all patients with GO [25, 26]. In the cases of moderate-to-severe GO, intravenous glucocorticoid therapy is the first line treatment [25]. Therefore, patient selection and treatment risks must be adequately addressed, since available therapies for GO are associated with potentially serious adverse effects [27]. This is an area where new treatment approaches are greatly needed. In previous papers, authors found an important role of different proangiogenic factors such as VEGF, PDGF, or TGFβ1 in the pathogenesis of GO and its correlation with clinical activity of the disease [5, 28–30].

It is known that HGF is a powerful inducer of angiogenesis with the activity compared to VEGF [31, 32]. Orbital inflammation in active GO is dominated by a T-helper 1 (Th1) cytokine environment with abundant production of cytokines such as IL-1β, IL-2, IFN-γ, and TNF-α and it was demonstrated that IL-1β stimulates orbital fibroblasts to produce Interleukin-8 (IL-8) which is a chemoattractant for B-lymphocytes, neutrophils, monocytes, and T-lymphocytes [33, 34].

The aims of this study were as follows: (1) to evaluate the serum concentration of HGF and IL-8 in the blood of newly diagnosed Graves’ disease patients with the first episode of active GO and healthy controls; (2) to estimate the influence of the thyroid function (euthyreosis vs. hyperthyreosis) on HGF and IL-8 blood levels in patients with active GO; (3) to evaluate the influence of intravenous methylprednisolone (MP) pulse therapy and additional oral MP therapy on HGF and IL-8 blood levels in patients with active GO.

The work was supported by the research programme (KNW-1-129/K/3/0) of the Committee of Scientific Research in Poland.

## Patients and methods

Thirty-nine Graves’ disease patients with the first episode of clinically active GO (Group A) were enrolled in the study. To estimate the influence of the thyroid function on serum concentrations of the studied proangiogenic factors, Group A was divided into Group A I (*n* = 18) in euthyroid and Group A II (*n* = 21) in hyperthyroid stage of Graves’ disease. The control group consisted of 20 healthy volunteers age- and sex-matched to the GO group [mean age 52.8 ± 7.6, min 34 max 64 years; male/female ratio: 5/15 (25/75 %)] without thyroid disease, autoimmune diseases, liver diseases, or history of immunosuppressive therapy.

All patients enrolled in the GO group were treated with anti-thyroid drugs (thiamazole) and mean duration after onset of GO was 4.6 months (min 1 month, max 7 months). Patients in the studied group were in moderate-to-severe stage of GO, according to the European Group on Graves’ Orbitopathy classification [25]. Epidemiological and clinical characteristics of the studied group of patients with active GO are presented in Table 1.

**Table 1:** Clinical characteristics of the patients with active Graves’ orbitopathy (GO) (Group A) and patients with GO in euthyroid (*n* = 18, Group A I) and hyperthyroid (*n* = 21, Group A II) stage of thyroid function before methylprednisolone treatment

	Group A (*n* = 39)	Group A I (*n* = 18)	Group A II (*n* = 21)
Age (years)	50 ± 9.4	51 ± 10.7	49 ± 8.4
Male/female	8/31 (20.5/79.5 %)	4/14 (28.6/71.4 %)	4/17 (23.5/76.5 %)
TRAb (IU/l)	16.776 ± 43.653	7.695 ± 14.726	24.559 ± 57.432
TSH (µIU/ml)	1.467 ± 3.375	3.121 ± 4.481	0.050 ± 0. 077
FT4 (ng/dl)	2.756 ± 7.088	1.168 ± 0.479	4.116 ± 9.542
CAS	5.692 ± 1.127	5.667 ± 1.328	5.714 ± 0.956
NO SPECS	4.079 ± 0.969	4.118 ± 0.781	4.048 ± 1.117

Values are mean ± standard deviation

*TRAb* TSH-receptor antibodies, *CAS* clinical activity score of GO, *NO SPECS* severity classification score of GO

The inclusion criteria for the study were the following: confirmation of Graves’ disease by clinical and laboratory findings [free thyroxine—fT4, TSH, TSH-receptor antibodies (TRAb) and, thyroid USG scans—enlargement of thyroid gland with typical diffuse increased vascularity] and ophthalmological examination confirming the active stage of GO (clinical activity score—CAS ≥3 points, magnetic resonance scans of the orbit). The exclusion criteria were any other causes of exophthalmos, other immune system disorders, other metabolic and infectious diseases, previous history of immunosuppressive therapy, and patients with malignant tumours.

All patients enrolled for the study were diagnosed and treated in Endocrinology Division, Department of Pathophysiology and Endocrinology, Medical University of Silesia and GO classification was made by consultant ophthalmologist (M.N.) with high experience in diagnosis and classifications of GO. In all patients the starting dose 0.5 g daily every other day, intravenous (i.v.) methylprednisolone (MP) infusion was maintained for the six infusions and then, in most cases, halved (0.25 g) for the remaining three or six infusions to the total maximum cumulative dose of 4.5 g (within 3 or 4 weeks). All patients obtained the middle cumulative dose of i.v.MP according to the recommendations of the EUGOGO [35]. The cumulative dose of i.v.MP depended on the clinical response to the treatment (ophthalmological assessment performed after six infusions). After i.v.MP therapy, treatment was continued with orally administrated MP in the initial dose of 24–32 mg daily (0.4 mg/kg of body weight) with dose reduction during the next 3 months.

Blood was collected after an overnight fast, and serum was obtained by centrifugation and it was stored at −70 °C. Concentrations of the studied proangiogenic factors in serum samples were measured by an enzyme-linked immunosorbent assay (ELISA) for human HGF (Ray Biotech, Inc., USA) and for IL-8 (Quantikine R&D System, USA), according to the manufacturer’s standard protocol before (Group A) and after (Group A1) intensive pulse i.v.MP treatment and 1 month after the end of additional oral MP treatment (Group A2). Intra- and interassay variations for HGF were 2.9 and 2.6 %, and for IL-8 5.6–6.5 and 6.1–7.4 %, respectively.

The statistical analysis was performed using the StatSoft Inc. (2011) Statistica v.10 data analysis software system. The distribution of the tested samples was determined by the Kolmogorov–Smirnov test. Kruskal*–*Wallis one-way analysis of variance with post hoc Dunn test was applied to compare the results in the studied groups of patients. For all statistical tests, a *p* value <0.05 was considered to indicate statistical significance.

All subjects gave their informed consent before participating in the study, and the study followed the tenets of the Declaration of Helsinki. The project was conducted with the approval of The Bioethics Board of the Medical University of Silesia (NN-6501-34/06, KNW/0022/KB/35/13).

## Results

**Fig. 1:**
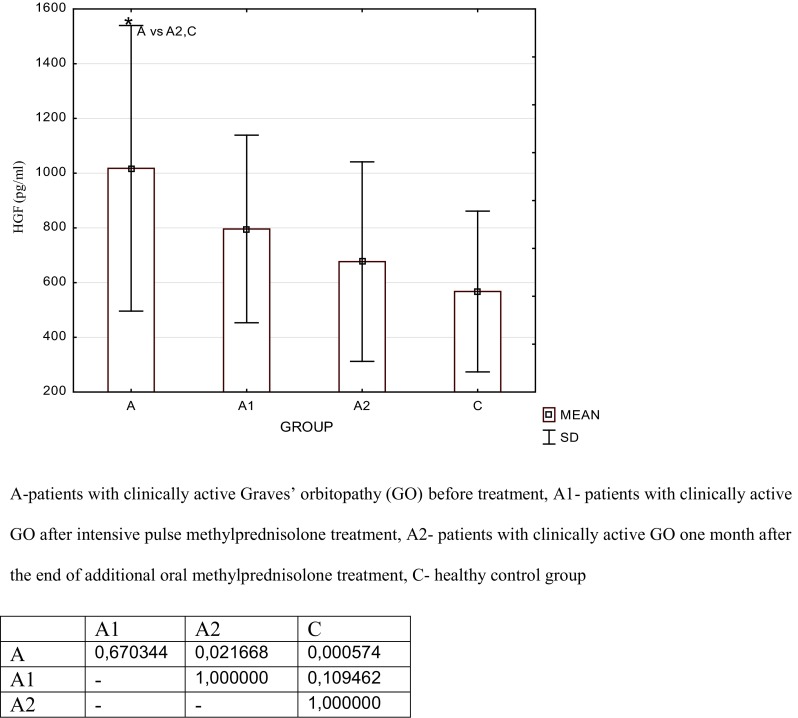
Serum concentration of hepatocyte growth factor (HGF, pg/ml) in the studied groups of patients and the significant differences table

**Fig. 2:**
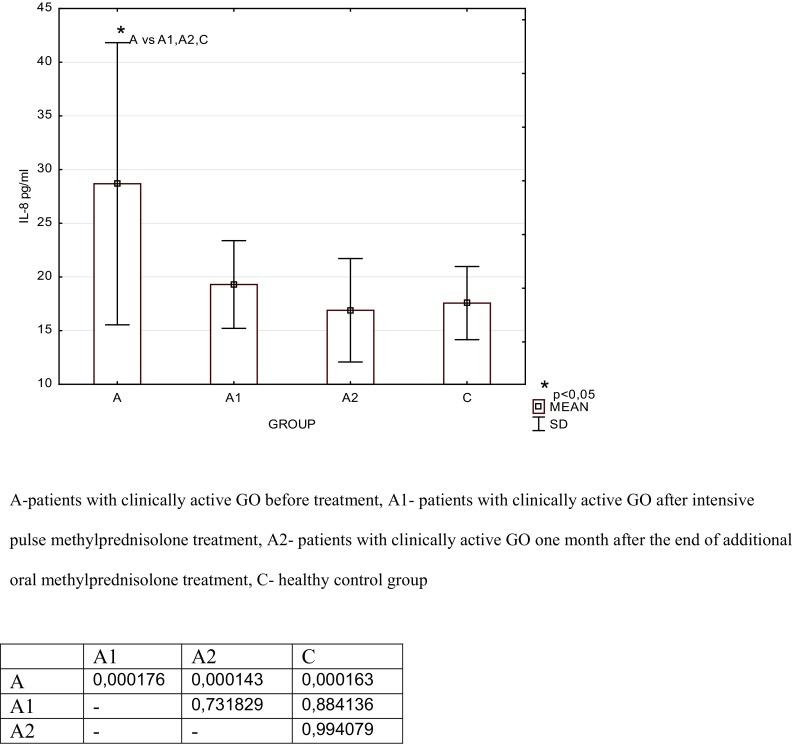
Serum concentration of interleukin 8 (IL-8, pg/ml) in the studied groups of patients and the significant differences table

The results of the study are presented in Tables 2 and 3 and in Figs. 1 and 2.

**Table 2:** Serum concentration of hepatocyte growth factor (HGF, pg/ml) in the studied groups of patients with active Graves’ orbitopathy and the healthy control group

Group	Mean	SD	Median	Minimum	Maximum	Lower quartile	Upper quartile
A	1035.0	465.0	933.0	312.0	3018.0	663.0	1284.0
A1	771.0	295.0	741.0	162.0	1452.0	582.0	1095.0
A2	678.0	345.0	582.0	216.0	1818.0	459.0	798.0
C	567.0	249.0	432.0	207.0	1203.0	342.0	813.0

*A* patients with clinically active Graves’ orbitopathy (GO) before intensive pulse methylprednisolone treatment, *A1* patients with clinically active GO after intensive pulse methylprednisolone treatment, *A2* patients with clinically active GO 1 month after the end of additional oral methylprednisolone treatment, *C* control group

**Table 3:** Serum concentration of Interleukin 8 (IL-8, pg/ml) in the studied groups of patients with active Graves’ orbitopathy and the healthy control group

Group	Mean	SD	Median	Minimum	Maximum	Lower quartile	Upper quartile
A	28.89	13.03	24.2	17.4	78.1	19.7	30.1
A1	19.29	4.08	17.9	14.6	29.1	16.9	21.2
A2	16.9	4.82	16.1	11.6	29.8	13.1	19.2
C	17.57	3.4	16.9	12.1	23.6	15.3	20.6

*A* patients with clinically active Graves’ orbitopathy (GO) before intensive pulse methylprednisolone treatment, *A1* patients with clinically active GO after intensive pulse methylprednisolone treatment, *A2* patients with clinically active GO 1 month after the end of additional oral methylprednisolone treatment, *C* control group

We found a significant increase in serum concentrations of the studied proangiogenic factors in the GO group before i.v.MP therapy when compared with the control group [HGF: 1035.0 ± 465.0 vs. 567.0 ± 249.0 pg/ml, *p* = 0.001; IL-8: 28.89 ± 13.03 vs. 17.57 ± 3.4 pg/ml, *p* = 0.0002]. After i.v.MP therapy, we found a significant decrease in IL-8 serum concentrations [19.29 ± 4.08 vs. 28.89 ± 13.03 pg/ml, *p* = 0.0002] and a decrease in HGF though not significant [771.0 ± 295.0 vs. 1035.0 ± 465.0 pg/ml, *p* = 0.670] as compared to the concentration before treatment.

One month after the end of additional oral MP treatment (Group A2), serum concentration of HGF decreased insignificantly compared to the concentration in Group A1 [A2: 678.0 ± 345.0 vs. A1: 771.0 ± 295.0 pg/ml; *p* = 1.000]. Serum concentration of HGF was significantly lower after the end of additional oral MP treatment (Group A2) when compared with the concentration in the GO group before i.v.MP therapy (Group A) [678.0 ± 345.0 vs. 1035.0 ± 465.0 pg/ml, *p* = 0.021]. After the end of additional oral MP treatment (Group A2), no significant difference was observed in HGF concentration when compared with the control group [678.0 ± 345.0 vs. 567.0 ± 249.0 pg/ml, *p* = 1.000]. Serum concentration of IL-8 also decreased after the end of additional oral MP treatment (Group A2) as compared with the concentration in Group A1 [16.9 ± 4.82 vs. 19.29 ± 4.08 pg/ml, *p* = 0.731], and we found no difference in the value between Group A2 and the control [17.57 ± 3.4 pg/ml, *p* = 0.994].

In the GO group before i.v.MP therapy, the mean CAS score was 5.718 ± 1.123 and we found a significant reduction in GO activity after i.v.MP (3.0 ± 1.138, *p* = 0.001) and a further significant reduction after the end of additional oral MP treatment (Group A2) (2.178 ± 1.403, A1 vs. A2 *p* = 0.011). Mean severity index of GO measured by the NO SPECS score in Group A was 4.077 ± 0.957 and decreased significantly after i.v.MP therapy (A1: 3.4 ± 0.976, *p* = 0.003). In Group A2, we also observed a reduction in the severity of GO (2.013 ± 1.276). We did not, however, find a significant difference as compared with the NO SPECS score in Group A1 (A2 vs. A1 *p* = 0.099).

We did not find the difference in serum concentration of the studied proangiogenic factors between patients in euthyroid and hyperthyroid stage of Graves’ disease before MP therapy (Table 4). Neither did we find any correlation between the studied proangiogenic factors and parameters of autoimmune thyroid disease such as TRAb.

**Table 4:** Serum concentration of hepatocyte growth factor (HGF) and interleukin-8 (IL-8) in the healthy control group (C) and in patients with active Graves’ orbitopathy in euthyroid (*n* = 18, Group A I) and hyperthyroid (*n* = 21, Group A II) stage of thyroid function before methylprednisolone therapy

Group	*n*	HGF (pg/ml)					*p*		
		Mean	SD	Median	Min	Max	C–AI	C–AII	AI–AII
C	20	567	294	432	432	1203	0.001	0.001	0.746
A I	18	969	363	923	345	1842			
A II	21	1101	585	1001	312	3018			

Group	*n*	Il-8 (pg/ml)					*p*		
		Mean	SD	Median	Min	Max	C–AI	C–AII	AI–AII
C	20	17.565	3.407	16.9	12.1	23.6	0.001	0.001	0.968
A I	18	43.49	74.954	24.1	17.4	367.1			
A II	21	31.724	15.354	26.5	18.3	78.1			

## Discussion

In our previous study, we had found a significant increase in serum concentrations of VEGF and PDGF-AA in the group with active GO before i.v.MP therapy when compared with the control group and a significant decrease in PDGF-AA and insignificant decrease in VEGF serum concentrations after i.v.MP therapy compared with the concentration before treatment. We had found that 1 month after the end of additional oral MP treatment, serum concentration of PDGF-AA decreased compared to the values after i.v.MP treatment and no significant difference was observed in the value between Group A2 and the control. However, serum concentration of VEGF was still significantly higher as compared to the control [5, 30].

In the present study, we found that serum from Graves’ patients with clinically active GO exhibited a “proangiogenic” profile, with enhanced serum levels of HGF and IL-8. Concentrations of HGF and IL-8 were significantly higher in the studied group before systemic immunosuppressive therapy when compared to the control. Furthermore, there was a decrease in the concentration of the studied proangiogenic factors after intensive MP therapy with a simultaneous decrease in the activity and severity of GO measured by CAS and NO SPECS score.

Our findings are consistent with several studies demonstrating an increase in serum concentration of proangiogenic factors in patients with Graves’ disease [5, 36, 37]. However, to the best of our knowledge, our study is the first to evaluate HGF concentration in Graves’ disease patients with clinically active GO.

Some authors found that serum VEGF level is elevated in patients with untreated Graves’ disease and correlates positively with the degree of thyroid vascularity assessed by colour Doppler ultrasound [37–39]. Other authors found elevated concentrations of PDGF, FGF, IGF-1, and TGF-β1 in patients with GO [28, 29, 38, 39].

Angiogenesis is critical for the growth and metastatic spread of tumours and chronic inflammation processes. Although HGF is well characterised as a hepatotrophic [40] and renotrophic factor [41] in liver and kidney regeneration, the presence of the local HGF system (HGF and its receptor—cMET) was demonstrated in both endothelial cells and vascular smooth muscle cells in vivo and in vitro [42]. Hepatocyte growth factor is produced by fibroblasts and other stromal cells and accumulates in the extracellular matrix and in basement membranes, bound to sulphated glycosaminoglycans [43, 44]. Serum level of HGF is a powerful inducer of angiogenesis [31]. Hepatocyte growth factor has an angiogenic action, as well as endothelium-specific growth action and the effect of HGF is reported to be stronger than that of VEGF [31, 45, 46]. Hepatocyte growth factor is characterised as an important mitogen (stimulation of cell growth), a motogen (stimulation of cell motility), and a morphogen (induction of multicellular tissue-like structure) [47, 48]. It is also a hypoxia-induced secreted protein that binds to cMET and regulates IL-8 expression.

The biological and clinical significance of HGF and its receptor cMET in the thyroid was studied by some authors [48–51]. Thus, overexpression of MET protein might represent an immunohistochemical marker of papillary carcinoma, potentially helpful in problematic cases [50]. MET protein is rarely observed in other thyroid tumours, including follicular carcinoma, anaplastic carcinoma, and medullary carcinoma [50, 51]. MET protein is not present in normal thyroid follicles, but a moderate expression of MET protein is observed in non-neoplastic thyroid diseases, such as Graves’ and Hashimoto’s thyroiditis [51], some cases of adenomatous goitre, follicular adenoma, and hyperfunctioning goitre [48], which is consistent with our study results.

Interleukin-8 was shown to be an angiogenic factor in 1992 [52]. It plays a significant role in cancerogenesis by mediating angiogenesis [53–57]. Additionally, IL-8 is implicated in a number of inflammatory diseases, such as cystic fibrosis [58], adult respiratory distress syndrome [59], chronic obstructive pulmonary disease, and asthma [60]. Interleukin-8 is secreted by multiple cell types, including monocytes, neutrophils, epithelial, fibroblast, endothelial, and mesothelial cells. It is also detected in tissues related to Graves’ disease, such as thyroid tissue, orbital fibroblasts, and the tears and blood of Graves’ patients [7, 61, 62]. Interleukin-8 is released from several cell types in response to an inflammatory stimulus [63–65] and is crucial for wound healing [66]. Interleukin-8 not only serves as a chemotactic factor for leukocytes and fibroblasts but it also stimulates fibroblast differentiation into myofibroblasts and promotes angiogenesis [67, 68]. Interleukin-8 is a chemoattractant for neutrophils and induces expression of several cell adhesion molecules and leads to neutrophil activation and hence it may contribute to the pathogenesis of inflammatory diseases [69, 70].

Interleukin-8 is also an oxidative stress-responsive proinflammatory chemokine, released from epithelial cells following particle-induced oxidative stress leading to neutrophil influx and inflammation [71, 72].

Orbital fibroblasts in patients with GO express elevated levels of CD40 ligand and it was found that CD40 ligand and TSH caused up-regulation of IL-8 concentration in orbital fibroblasts of GO patients compared to controls [61, 73].

Huang et al. found higher concentrations of IL-8 in the tears of Graves’ disease patients with GO compared to Graves’ disease patients without GO [62]. The concentration of IL-8 was also increased in the orbital adipose tissue in patients with Graves’ disease and GO compared to control patients with Graves’ disease without GO [7]. These findings suggest that IL-8 may be a mediator of GO activity. Douglas et al. confirmed that stimulation of both TSHR and CD40 plays a role in IL-8 production in circulating fibrocytes. These authors concluded that such stimulation may be a significant pathway for IL-8 production in Graves’ disease and GO [74].

In our study, we found a significant increase in IL-8 in the GO group when compared with the control group and a significant decrease after i.v.MP therapy, which is consistent with the results of other authors. One month after the end of additional oral MP treatment, serum concentration of IL-8 still decreased and we did not find the difference with the value in the control group.

Our study supports the observations of Matos et al. who found that patients with a positive expression of proangiogenic factors (FGF, IGF-1, and VEGF) in extraocular eye muscles showed CAS greater than 5, thereby suggesting an important role of these growth factors in the pathogenesis and severity of GO [39].

Glucocorticoids (e.g. MP) modulate VEGF production (and probably also other proangiogenic growth factors) and Ye et al. found that VEGF levels decreased in patients with GO after glucocorticoid treatment, and these changes were accompanied by a decrease in CAS. According to these authors, this decrease could reflect the degree of orbital inflammatory activity [29].

Recently, authors have demonstrated elevated PDGF mRNA expression in the orbital tissue from patients with GO [75]. In other studies, authors found that PDGF-BB induces the production of IL-6, IL-8, CCL2, CCL5, and CCL7 by orbital fibroblasts. The above cytokines are involved in the pathogenesis of GO [64]. It is obvious that pathological angiogenesis occurs in inflammatory diseases. The identification of sensitive and specific markers as well as the molecular basis and pathways of diseases with increased angiogenesis should be a challenge for further research. A better understanding of the process may also enable the design of efficient antiangiogenic therapy.

## Conclusions

1. Serum HGF and IL-8 concentrations are elevated in Graves’ disease patients with active Graves’ orbitopathy as compared to the healthy control group.
2. The elevated serum HGF and IL-8 in patients with active Graves’ orbitopathy may reflect long-standing autoimmune inflammatory processes in orbital and thyroid tissues and intensified angiogenesis.
3. Successful management of active Graves’ orbitopathy with glucocorticoids is associated with a decrease in HGF and IL-8 serum concentrations.
4. Examination of the proangiogenic factors in the blood of patients with Graves’ orbitopathy (in connection with clinical signs measured by CAS and orbit MR scans) could be one of the parameters in the evaluation of Graves’ orbitopathy activity and response to the immunosuppressive treatment, however, further studies are still required.
